# Prevalence, Circumstances, and Risk Factors of Falls Among Community Dwelling Members of University of the Third Age

**DOI:** 10.3389/fpubh.2021.610504

**Published:** 2021-11-24

**Authors:** Asmidawati Ashari, Tengku Aizan Hamid, Mohd Rizal Hussain, Rahimah Ibrahim, Keith D. Hill

**Affiliations:** ^1^Department of Human Development and Family Studies, Faculty of Human Ecology, University Putra Malaysia, Serdang, Malaysia; ^2^Laboratory of Social Gerontology, Malaysian Research Institute on Ageing MyAgeing™, University Putra Malaysia, Serdang, Malaysia; ^3^School of Physiotherapy and Exercise Science, Curtin University, Perth, WA, Australia; ^4^School of Primary and Allied Health Care, Monash University, Melbourne, VIC, Australia

**Keywords:** accidental falls, risk factors, community dwelling, older adult, balance

## Abstract

**Objective:** Study aimed to identify the prevalence of falls and associated contributory factors among older Malaysians.

**Methods:** A cross sectional study among community dwelling older adults aged 50 years and above. Self-administered questionnaires on history of falls in the previous 12 months, physical assessment and computerized and clinical measures of balance were assessed on a single occasion.

**Results:** Forty nine (31.0%) participants fell, with 4.4% reported having multiple falls within the previous 12 months. Slips were the most prevalent cause of falls, accounting for 49% of falls. More than half (54.5%) of falls occurred in the afternoon while participants walked inside the home (32.7%), outside home (30.6%), and 36.7% were in community areas. More than half of respondents were identified as having turning instability. Step Test, turn sway, depression, physical activity level and edge contrast sensitivity were significantly worse for fallers (*p* < 0.05). Multiple logistic regression analysis showed that turning performance, visual acuity and back pain were significantly associated with falls risk, accounting for 72% of the variance of risk factors for falls among studied population.

**Conclusion:** Falls are common among community dwelling older Malaysians. The findings provide information of falls and falls risk factors among community dwelling older adults in Malaysia. Future intervention studies should target locally identified falls risk factors. This study has highlighted the importance of instability during turning as an important fall risk factor.

## Introduction

Falls are common events among older people and have become known as one of the “geriatric giants” (1–3). Due to their increasing falls prevalence with increasing age (4) and the unprecedented rate of aging populations worldwide (5–7), falls have become one of the most common and serious health problems in older adults (8–11). A recent review of epidemiological studies of fall across countries, indicated that the range of fall in the community setting older person is between 20 and 33%. These falls resulted in serious injuries in 10–20% of cases, and 2–6% resulted in a fracture or other serious injuries requiring hospitalization.

Although there are many studies of falls risk factors and falls prevention interventions, the majority of these have been undertaken in Western countries (1, 12). There have been a small number of studies that have reported falls incidence in Asian nations such as Taiwan (8, 13), Japan (14, 15), Korea (16–18), Hong Kong (13), Singapore (19, 20), and Thailand (21). These small number of studies have reported varying rates of falls, between 10 and 33%, for the proportion of older people falling in Asian countries in a 12 month period (7). Differences in age, racial background, socio-demography, environment and population settings, life-style (diet, activity, sunlight exposure), body dimensions, and culture between countries might all contribute to the differences of fall prevalence, circumstances and consequences of falls across different regions of the world (22–24). Additionally, differences in sampling, recruitment and methods of data ascertainment (retrospective vs. prospective falls data), and possibly cultural differences in willingness to report specific health problems such as falls, may account for some of these differences. In addition, research is needed to identify prevention strategies that will be effective in different cultural contexts (3, 25).

The older population in Malaysia is growing rapidly. In 2007, they were 1,195,480 people aged 65 years and above which represented 4.41% of the total population (Department of Statistic, 2018). It was projected that by the year 2019, older persons population aged 65 and above would reach 7% and would double to 14% in 2043 and Malaysia will become and aged nation by year 2030 whereby the older population comprises 15% of the total population. This demographic change seen older people forming a more significant part in the Malaysian society. This situation creates the need for preventive action to minimize the impact of the problems associated with aging, such as falls. Therefore, the magnitude of the problem of falls and associated contributory factors needs to be defined in the Malaysian context, and management strategies should be designed within the context of local needs and the Malaysian primary and public health care systems. The aims of this study were to identify the prevalence of falls in community dwelling older Malaysians, and associated risk factors and circumstances.

## Methods

### Study Design

A cross-sectional study using convenience sampling was undertaken to quantify the prevalence of falls in previous 12 months and to identify the risk factors for falls and circumstances of falls among studied population. In the present study, a fall was defined as “unintentionally coming to the ground or some lower level and other than as a consequence of sustaining a violent blow, loss of consciousness, sudden onset of paralysis as in a stroke or epileptic seizure”^1^.

### Setting, Participants and Sampling

Participants were recruited through the distribution of flyers to all members of University of the Third Age (U3A) Selangor and Kuala Lumpur, Malaysia, based at the Institute of Gerontology, Universiti Putra Malaysia. University of the Third Age (U3A) Malaysia, is program that provide lifelong learning courses to older adult aged 50 years and above. Inclusion criteria for the study were (i) living in the community and (ii) being aged 50 years and above. The cut-off age of 50 years and above was used in this study rather than 65 years of age because of a lower life expectancy in Malaysia compared to in Western countries. The life expectancy for Malaysia was 72.2 and 77.3 years for males and female, respectively (26), and (iii) able to tolerate standing at least 6 min independently or with a single point stick support. Older adults who expressed interest in participation were contacted through an initial phone call to discuss details of what the project involved. Screening was carried out during this phone conversation to ensure all eligibility criteria for the study were met. Written consent was obtained from each participant.

Data were obtained by trained researchers based on a structured questionnaire, and a series of tests of physical performance. The structured questionnaire comprised of five main sections: (i) socio-demography information, (ii) physical and medical conditions, (iii) history of falls and falls risk assessment, (iv) psychological aspect associated with falls, and (v) self-reported level of physical activity. The physical performance measures, reported below, included both clinical and laboratory assessments of balance and mobility. The questionnaires were interspersed between the physical performance tests. The interview and assessments were conducted at the assessment laboratory and lasted ~90 to 120 min. Participants were allowed to have a rest between the tests if required.

### Measurements

Height (SECA Bodymeter), weight and body composition (BF-418, TANITA, Japan) were measured by trained assessor. Level of physical activity was assessed using Human Activity Profile, a questionnaire with 94 activity items listed in order of increasing energy expenditure. Each item was rated as “still doing,” “have stopped doing,” or “never did.” The Adjusted Activity Score (AAS) (highest numbered item listed as “still doing” less the number of items rated as “have stopped doing”) was used in the analysis.

Visual edge contrast sensitivity was assessed using the Melbourne Edge Test. This test presents 20 circular patches containing edges with reducing contrast (27, 28), with the highest numbered circle where the direction of the edge of contrast is correctly identified recorded and were reported in the study.

#### History of Falls and Fall Risk Assessment

Participants were asked to recall any falls in the preceding 12 months. For each fall, data was reported on location, activity at the time of fall, obstacles involved in the fall, any warning signs, type of injuries and medical attention sought (falls circumstances were collected for up to a maximum of four falls). Falls risk was assessed using the Falls Risk Assessment of Older Person-Community version (FROP-COM), a validated tool with moderate accuracy in predicting future falls (29).

#### Clinical Measures of Balance and Mobility

The clinical test battery used in this study included simple and quick tests of balance performance that are routinely used in clinical practice and research and been shown to be reliable and valid in older populations as followed; Timed Up and Go (TUG) Test (30, 31), The Functional Reach (FR) test (32), The Step Test (ST) was used to evaluate the speed of performing a dynamic single limb stance task (33).

#### Computerized Balance Assessment

Computerized Postural Balance Performance tests were undertaken using Neurocom® Balance Master force balance platform (Neurocom International, Inc., Clackamas, OR, USA), which computes forces from force transducers under the plate on which the participant stands or moves (34, 35). Participants wore a safety harness attached to an overhead rail for the first two tests. There are five measures of balance performance were assessed on the Balance Master long force plate as follows; (i) Modified Clinical Test of Sensory Integration of Balance (mCTSIB); (ii) Limits of stability (LOS) test were used to quantify the ability of the participant to intentionally displacing their Center of Gravity (COG). The LOS measure has been shown to be sensitive to identify fallers and predict future falls and to have good internal consistency, ICC > 0.84; (iii) Sit to Stand (STS) test was performed on the long force plate to quantify several movements characteristics related to the ability to stand up from a seated to a standing position without overbalancing; (iv) Walk Across (WA) test was used to quantify several characteristics of gait including stride length (cm) and step width (cm); and (v) The step quick turn (SQT) test was used to quantify two movement characteristic as the participant quickly turned 180 degrees. All test was repeated three times and the average was used for data analysis.

#### Psychological Measures

Psychological aspects associated with falls (fear of falling and depression) were measured using the Short Falls Efficacy Scale-International (FES-I) (36) and the Geriatric Depression Scale (GDS-15) (37), respectively. The FES-I measured the participants' self-reported level of concern about falling when performing seven selected activities. The score was ranged from 1 (not concern at all) up to 4 (very concern) for each item, with a maximum score of 28.

### Statistical Analysis

Descriptive analysis was applied to participant demographic data and all outcome measures. Performance on all outcome measures were compared between participants who reported one or more falls in the preceding 12 months and those reporting no falls, using *t*-test for continuous/normally distributed variables, and chi squared for categorical variables. Univariate logistic regression was performed to determine variables associated with falls and variables with a *p* < 0.1 were then included in a multivariate logistic regression (38). All analyses were conducted using SPSS vs. 22.0, and the critical value for all analyses was *p* < 0.05.

## Results

### Participants Profile

A total of 156 participants were assessed (Table 1). The age of study participants ranged from 50 to 78 years old, with mean (SD) age was 63.2 (6.2), and more than half of the participants (57%) were females. The majority of participants were Malay (83.4%) and most were still married (76.3%). In terms of education level, all participants were literate (able to write and read) with more than half of participants (56.5%) having received secondary education. Most participants (73.1%) were retired and the median income for study participants was MYR1900 per month. There was no significant difference of socio-demographic between faller and non-faller.

**Table 1:** Socio demographic profile of respondents (*N* = 156).

**Variables**		**Means (SD)** ** Median [IQR]**	**Percentages (%)**
Sex	Male		43.1
	Female		56.9
Age (years)		63.2 (6.2)	
Ethnicity	Malay		80.6
	Non-Malay		19.4
Marital status	Married		76.3
	Divorced/widowed/ Never Married		23.7
Education level	Primary		13.5
	Secondary		54.5
	Tertiary		32.0
Living arrangement	Lived alone		6.4
	Lived with spouse only		26.3
	Lived spouse/children/ others		67.3
No. of children		3.6 (2.1)	
Monthly Income (MYR)		MYR1900 [MYR2000]	
Employment status	Not working/ retirees		89.4
	Still working		10.6
Cognitive score (AMTS)		9.44 (0.8)	

*M, mean; SD, standard deviation; Mdn, median; IQR, interquartile range; MYR, Malaysian Ringgit; AMTS, Abbreviated mental Test Score*.

### Health Condition and Psychological Factors

In terms of health and medical conditions, 56% of participants reported of having at least one to three health problems and 7.5% had four and more health problems that might affect their balance performance (Table 2). The most common health conditions reported were diabetes mellitus (19.2%), arthritis (16.7%), cardiac problems (14.7%), and back pain (14.7%). Thirty percent of participants reported taking three or more types of medication daily and antidepressant (29.5%) was the most common taken medication. Only two participants in present study, used a single point of stick during outdoor activity. The majority of participants were considered not to have depressive symptoms based on a GDS score of between 0 and 4 (39, 40), with an overall mean score for GDS-15 of 2.22 ± 2.3 SD. Study also found that the mean score of FES-I was 17.6 (6.1 SD) and 16.8 (6.4SD) among non-fallers and fallers respectively.

**Table 2:** Physical and health profile of participants (*N* = 156).

**Variables**	***M* (SD)/*Mdn* [IQR]**	**Percentages (%)**
Height (cm)	155 (8.1)	
Weight (kg)	66.2 (13.3)	
**BMI (kgms** ^**−1**^ **)**		
Underweight (<18.5)		0.8
Normal (18.5–24.9)		23.1
Overweight ≥ 25		48.1
Obese		28.0
**Number of health conditions**		
None		36.1
1–3 conditions		56.4
4 or more conditions		7.5
**Medical problems**		
Diabetes mellitus		30 (19.2)
Arthritis		26 (16.7)
Cardiac problems		23 (14.7)
Back pain		23 (14.7)
Dizziness		19 (12.2)
Neurological condition		19 (12.2)
Respiratory problem		13 (8.3)
Osteoporosis		7 (4.5)
**Number of prescribed medications**		
No medication		36.1
1–2 medications		33.8
3 or more medications		30.1
Fall Efficacy Score (FES-I)	17.6 (6.1)	
Geriatric Depression Scale (GDS-15)	2.1 (1.9)	

*M, mean; SD, standard deviation; Mdn, median; IQR, interquartile range; BMI, Body Mass Index*.

### Prevalence, Circumstances, and Consequences of Falls

Study found 49 (31.4%) participants had at least one fall in the previous 12 months, and seven of these participants (14.3%) reporting experiencing two falls within the 12 months prior to the date of interview (Table 3). Out of the 49 participants who fell, 31 (63.3%) were female and 18 (36.7%) were males. The mean age of fallers and non-fallers were 63.3 years (6.5 SD) and 62.5 years (6.8 SD), respectively. There was no difference in the proportion of fallers who were male (36.7%) or female (63%), χ^2^ (1, *n* = 156) = 1.36, *p* = 0.243 nor between the three age groups (36.7%) of fallers were aged 50–59, 32.7% aged 60–69 and 30.6% aged 70 years and above, χ^2^ (2, *n* = 156) = 1.36, *p* < 0.05.

**Table 3:** Falls related information (*n* = 49).

**Information of fall**		**Frequency (Percentage %)**
Number of falls within last 12-month period	One	42 (85.7)
	Two	7 (14.3)
Cause of falls	Trip	15 (30.5)
	Slip	23 (46.9)
	Loss of balance	11 (22.4)
Location of falls	Inside home	17 (34.7)
	Outside home	14 (28.6)
	Community area	18 (36.7)
Time of falls	Day time	23 (46.9)
	Night time	26 (53.1)
Activity during falls	Walking	23 (40.8)
	Turning	20 (46.0)
	Bending/reaching/avoiding obstacle	6 (12.2)
Experience falls related injuries	No injury	9 (18.3)
	Minor Injury (did not require medical attention)	22 (44.9)
	Minor Injury (did require medical attention)	13 (26.5)
	Severe injury (fracture, dislocation, hospitalization)	5 (10.2)

The self-perceived causes and consequences of fall were obtained from the participant's explanation regarding their falls. Most fallers fell at home (63.3%). The bathroom, kitchen and dining area, and bedroom were the most common areas where falls occurred inside the home, and the backyard area was the most prevalent area reported for falls occurring outside the home. Thirty seven percent of fallers reported falling in community areas, with the most frequent location for these falls being in front of shops, streets (including curb, uncovered drain or uneven walking paths), and park areas. Trips and slips were the most prevalent causes, accounting for 77% of falls, with a further 20.4% being described as due to loss of balance. Almost a third of fallers fell in the forwards direction and 18.4% fell backward. Based on self-report regarding the severity of injuries incurred during falls, nine fallers (18.4%) reported no injury, while 40 fallers (81.6%) suffered injuries from their fall. More than half of fallers (71.4%) sustained minor injuries such as grazes, bruises, sprains, and cuts, while 10.2% reported suffering a severe injury such as fracture or dislocation as a consequence of their fall. Among those five participants who experienced severe injuries, one participant had a fractured patella, one experienced an ankle fracture, one had a wrist fracture and two had ankle dislocations. Meanwhile, those fallers who had minor injuries, 22 (62.8%) did not require medical treatment and 37.2% did seek medical attention. Consequently, most of the fallers perceived these injuries has restricted their mobility for at least 3 days up to a month.

### Risk Factors for Falls

Independent group *t-*test indicates a number of significant differences were identified between fallers and non-fallers with fallers having higher BMI, lower MET score, and higher depression score (Table 4), as well as poor balance (Step Test), and more sway and took longer time to perform the step quick turn test, and slower walking speed (Table 5). In addition, presence of back pain was significantly associated with falls status, χ^2^(1) = 3.56 (*p* ≤ 0.05). Participants with those reporting back pain are three time odd to fall than those without back pain (O.R: 2.87).

**Table 4:** Univariate analysis of physical and psychological condition of participants according to fall status.

**Parameter**	**Non-faller** ** (*n* = 107) Mean (SD)**	**Faller (*n* = 49) Mean (SD)**	***P*-value**
Body mass index (kg/ms^−2^)	27.2 ± 4.7	28.6 ± 4.5	0.071^*^
Contrast sensitivity (MET score)	21.2 ± 1.7	20.5 ± 1.9	0.028^**^
**Physical activity and psychological aspect**			
Fall efficacy score (FES-I)	17.6 ± 6.1	16.8 ± 6.4	0.437
Depression (GDS-15)	1.9	2.9	0.008^**^
Physical activity level (HAPAAS)	65.4	62.7	0.328

* *statistically significant at p < 0.05*,

** *statistically significant at p < 0.01*.

**Table 5:** Univariate analysis of balance and mobility performance of participants by fall status.

**Parameter**	**Non-faller (*n* = 107) Mean (SD)**	**Faller (*n* = 49) Mean (SD)**	***P*-value**
**Clinical measures of balance**			
Functional reach test	27.4, 6.5	28.0, 5.7	0.577
Step test (worst side)	16.5, 3.5	14.9, 3.7	0.012^**^
Timed up and go	10.1, 1.9	10.6, 2.3	0.177
Timed up and go with dual task	11.3, 2.5	12.2, 3.6	0.066
Timed chair stands	17.4, 5.9	17.6, 5.4	0.873
**Laboratory measures**			
mCTSIB—mean COG sway (deg/sec)	0.6, 0.4	0.7, 0.4	0.331
LOS—composite movement velocity (deg/sec)	3.1, 1.3	2.9, 1.0	0.223
LOS—composite reaction time (sec)	1.9, 1.2	1.3, 1.3	0.552
Walk across—step width (cm)	17.7, 6.2	17.8, 3.0	0.853
Walk across—speed (cm/sec)	62.8, 15.0	57.4, 16.8	0.044^**^
Sit to stand test- mean rising index (%of body weight)	16.2, 8.5	13.8, 5.8	0.073
Sit to stand test- mean COG sway (deg/sec)	2.8, 0.9	2.8, 0.9	0.801
Step quick turn—worst side time turning (sec)	2.2, 0.8	2.5, 0.8	0.039^**^
Step quick turn worst sway turning (deg/sec)	49.0, 8.0	52.2, 9.3	0.028^**^

** *statistically significant at p < 0.01*.

A multiple logistic regression was performed to ascertain the effect of BMI, step test, turning performance, degree of sway and turn taken during step quick turn test, level of depression, having back pain problem and edge contrast sensitivity on the odd that participants have fall as listed in Table 6. The logistic regression model was statistically significant, χ^2^(3,7) = 7.885, *p* = 0.048. The model explained 20.7% of the variance in having a fall, and correctly classified 71.8% of fallers. Those who were identified as having imbalance during turning were 4.8 times more likely to have a fall compared to those who did not have turning impairment.

**Table 6:** Multiple logistic regression association with fall status.

**Parameter**	**Exp (B)**	**95% CI**		***P*-value**
		**Lower**	**Upper**	
BMI (Kgms^−2^)	1.065	0.977	1.166	0.156
Visual contrast sensitivity (MET score)	0.874	0.701	0.809	0.028^*^
Depression (GDS score)	1.149	0.938	1.408	0.179
Back pain (no)	0.214	0.077	0.596	0.003^**^
Timed up and go (dual task)	0.941	0.759	1.167	0.579
Walk across—speed (cm/sec)	1.010	0.974	1.047	0.072
Step test (worst leg)	0.888	0.845	1.070	0.607
Turning impairment (yes)^#^	4.824	1.707	12.839	0.003^**^

* *Statistically significant at p < 0.05*,

** *Statistically significant at p < .01*.

*OR, odd ratio; CI, confidence interval; BMI, Body mass Index; MET, Melbourne Edge Test*;

# *Turning impairment categorized—yes = turn sway or turn time or both outside of normal limits on the Neurocom™ Balance Master*.

## Discussion

Results from this study indicated the frequency of falls among older people in Malaysia was common and comparable to previous a range of 13.8 to 62.1% as reported by other studies (41). Of note, the majority of Asian studies reporting falls rates were substantially lower than those reported in the present study. Our study, similar to the majority of studies reported in the Kwan et al. (13) review used retrospective recall of falls in the past 12 months, which has been shown to under-estimate actual falls numbers by ~20% due to the possibility that participants has been forgotten to recall their fall experiences (42). There are other factors that might contribute to the lower falls rates reported in some of these previous studies, including not having a clear definition of fall and different interpretation between participants and researcher. Zecevic et al. (43) concluded that older people prone to consider falls resulted from loss of balance, due to the unintentionally trip or slip whereas healthcare professional tend to refer falls as incident that leads to injuries and illness. In cultural perspective, among Asian population, fall was seen to be disgraceful experience that might be reported to other people (44). Interestingly, the current study found that those in the group 50's experienced more falls compared to older group. This finding is similar to previous study which reported that 62% of those were aged 50 years and above experienced more fall compared to others older age groups (41) and consistent with the other Asian studies that reported the younger age groups experienced more falls (16). Possible explanations for this unexpected finding may be due to the recruitment which constituted participants among U3A membership that relatively healthy and active. Furthermore, the younger group may be involved in more vigorous activity levels that could increase exposure to the risk of falls as compare to older aged group that less active.

In contrast to the previous studies, there is no significant effect of age, gender and socio demographic aspect contribute the prevalence of fall in the present study. Variables that significantly correlated with falls were: step test, edge contrast sensitivity score, BMI, having back pain, depression level and abnormal turning performance during SQT test. The most significant variable for fall was instability during turning with odds ratio 4.82.02 increasing risk of falling. As noted, majority of the participants have been classified of having turning instability based on the measures of Neurocom™ Step quick turn Test. Step quick turn is one of the computerized protocol that provides objective measure of turn sway and turn time (aged and gender matched) normal limit were found to be significantly correlated to falls among studied population. This finding informed that turning was a major problem in the studied population. Concurrently, previous finding reported that fall during turning may resulted eight times of hip fractures (4). Therefore, possible intervention that address on improving turning performance could be benefit to the studied population.

Nonetheless, there is a potential bias in using a volunteer sample, as it may not accurately represent the overall community dwelling population especially comparing the current findings with other study that used representative sample. In addition, the aged of 50 years and above was used in this study which differed compared to others from the previous report which applied cuts off age of 60 years and above.

## Conclusion

The findings of this study provide insight into the circumstances, consequences and correlates risk of falls in the Malaysian context, and highlight that fall is a common problem among active older adult community dwelling Malaysians. Results also highlighted that one of the main factors differentiating fallers from non-fallers that has rarely been investigated in the past is unsteadiness during turning activities, and this risk factor warrants further investigation in terms of contributory factors and potential interventions.

## Data Availability Statement

The original contributions presented in the study are included in the article/supplementary material, further inquiries can be directed to the corresponding author/s.

## Ethics Statement

The studies involving human participants were reviewed and approved by Research Ethics Committee of University Putra Malaysia (UPM/FPSK/PADS/T7-JK Etika-PerF01-Nov/10/Q1). The patients/participants provided their written informed consent to participate in this study.

## Author Contributions

AA, TH, and KH conceived the study and participated in the study design, measurement and assessment protocol, and drafted the manuscript. MH and TH participated in formal analysis of data and drafted the manuscript. RI participated in drafting the manuscript. All authors reviewed and approved the final version of the manuscript.

## Funding

This research was funded by Putra Grant Scheme, Universiti Putra Malaysia (GP-IPM/2013/9404500).

## Conflict of Interest

The authors declare that the research was conducted in the absence of any commercial or financial relationships that could be construed as a potential conflict of interest.

## Publisher's Note

All claims expressed in this article are solely those of the authors and do not necessarily represent those of their affiliated organizations, or those of the publisher, the editors and the reviewers. Any product that may be evaluated in this article, or claim that may be made by its manufacturer, is not guaranteed or endorsed by the publisher.

## Footnotes

^1^The prevention of falls in later life. A report of the Kellogg International Work Group on the Prevention of Falls by the Elderly. (1987). Danish Medical Bulletin, 34 Suppl 4, 1–24.
